# Play Together? Unveiling Facilitators and Barriers to Inclusive Outdoor Play for Dutch Children With and Without Disabilities: A Qualitative Study

**DOI:** 10.1111/cch.70154

**Published:** 2025-09-29

**Authors:** R. Q. Beekhuizen, E. A. M. Bolster, J. W. Gorter, N. L. Henry, K. Visser, H. Wittink, E. M. W. Kotte, M. E. Sol, M. A. T. Bloemen

**Affiliations:** ^1^ Research group Lifestyle and Health/Moving, growing and thriving together University of Applied Sciences Utrecht Utrecht the Netherlands; ^2^ Research group Lifestyle and Health/Moving, growing and thriving together, Institute of Human Movement Studies, Master Paediatric Physiotherapy University of Applied Sciences Utrecht Utrecht the Netherlands; ^3^ Department of Rehabilitation, Physical Therapy Science and Sports, UMC Utrecht Brain Center University Medical Center Utrecht Utrecht the Netherlands; ^4^ De Hoogstraat Rehabilitation Utrecht the Netherlands; ^5^ Department of Human Geography and Spatial Planning, Faculty of Geosciences Utrecht University Utrecht the Netherlands; ^6^ Research group Lifestyle and Health University of Applied Sciences Utrecht Utrecht The Netherlands; ^7^ Fitkids Foundation Amsterdam the Netherlands

**Keywords:** disability, inclusion, outdoor play, paediatric rehabilitation, qualitative research

## Abstract

**Background:**

The ‘Right to Play’ is included in the United Nations Convention on the Rights of the Child. Outdoor play contributes to children's overall development, physical and mental health and quality of life. Unfortunately, children with disabilities often experience restrictions while playing outdoors. Understanding children's perspectives is crucial to effectively support them in participating. Our aim was to explore facilitators and barriers perceived by Dutch children with and without disabilities (6–12 years), in regular and special primary education, for participating in inclusive outdoor play.

**Methods:**

We conducted 40 semi‐structured interviews in school‐aged children (mean age years 8.7, SD 1.9): 22 without disabilities, 10 with disabilities attending regular education and 8 with disabilities attending special education. We transcribed all interviews verbatim, and two independent researchers analysed the data using an inductive thematic approach.

**Results:**

We identified three main themes: personal factors of children with and without disabilities, interacting factors and environmental factors. Each theme has sub‐themes acting as either facilitators or barriers to inclusive outdoor play. Personal factors include being open to playing with each other, coping with disabilities, experiencing autonomy, insufficient knowledge about disabilities and feelings about physical and social limitations. Interacting factors include growing up together, making contact and adapting ways of playing. Environmental factors include parents, play environments, communities and time constraints.

**Conclusions:**

Children with and without disabilities in regular and special primary education identify facilitators and barriers in personal, interacting and environmental factors for inclusive outdoor play. Children with disabilities encounter more barriers than those without disabilities. All children are open to playing together, and paediatric rehabilitation professionals and parents play a crucial role in facilitating positive play experiences early on, providing knowledge about the consequences of disabilities, showcasing the capabilities of children with disabilities and collaborating to adapt playgrounds.

## Summary

- To our knowledge, our study is the first to explore the perspectives of both Dutch children without disabilities and children with disabilities in regular primary education and special primary education about inclusive outdoor play.
- Children with and without disabilities in regular and special primary education indicate facilitators and barriers in personal, interacting and environmental factors for inclusive outdoor play.
- Children with disabilities encounter more barriers than children without disabilities, particularly those in special primary education facing most barriers.
- The ‘Right to Play’ is protected in the United Nations Convention on the Rights of the Child, and paediatric rehabilitation professionals, with parental support, play a crucial role in fostering inclusive outdoor play for children.

## 1 Introduction

The ‘Right to Play’ is protected in the United Nations Convention on the Rights of the Child, and the importance of outdoor play for children is increasingly recognized (United Nations General Assembly 1989). Through play, children interact with their environment, develop social and emotional skills, form friendships and decrease the risk of social isolation (de Vries 2012; Fjørtoft 2001; Gill 2014; Nijhof et al. 2018; Vanderschuren and Trezza 2014). However, there is an alarming decline in the number of Dutch children who regularly engage in outdoor play. In 2013, 19% of Dutch children played outside less frequently than they desired, and this percentage increased to 44% in 2022 (Kaal 2022). This is concerning, as outdoor play contributes to a physically active lifestyle and positively impacts children's quality of life (Donnelly et al. 2016; Eime et al. 2013; Janssen and Leblanc 2010; Keawutan et al. 2014; Lankhorst et al. 2021; Maher et al. 2015).

Children with disabilities encounter more barriers to outdoor play compared to children without disabilities, resulting in less outdoor playtime (Burghard et al. 2018). These barriers can manifest in both the physical and social context, such as inaccessible playgrounds or exclusion by other children (Barron et al. 2016). In 2019, the Dutch Ministry of Health, Welfare and Sport (VWS), together with 12 Dutch organizations active in supporting inclusive outdoor play, endorsed ‘Het Samen Speel Akkoord’ (Play Together Agreement). Their aim was to establish an inclusive outdoor play culture, increase the number of inclusive play areas and develop knowledge about inclusive outdoor play (SamenSpeelAkkoord 2019). The term ‘inclusive’ refers to the participation of every child, regardless of disability status, in outdoor play, emphasizing the importance of play for all children (International Play Association 2016).

Parents as well as health and welfare professionals are important intermediates in reducing barriers and supporting inclusive outdoor play (Van Engelen et al. 2021). Professionals can implement behavioural interventions for children, aiming to facilitate interaction between children with and without disabilities, and empower parents (Bolster et al. 2021). Parents and professionals recognise the importance of inclusive outdoor play and emphasise the need for more practical knowledge and tools to effectively support children (Van Engelen et al. 2021). Interestingly, children's perspectives about inclusive outdoor play remained underexplored (Morgenthaler et al. 2023). Understanding facilitators and barriers for inclusive outdoor play experienced by children with and without disabilities is essential to provide effective support in playing together outdoors. Moreover, children's perspectives may differ from adult perspectives.

In the Netherlands, children with disabilities often attend regional special primary education (SPE) rather than local regular primary education (RPE). Most children attending SPE use taxi transportation to travel to school. Taxi transportation is time consuming; most children leave early in the morning and return home late in the afternoon, leaving little time to play outdoors (Verschuren et al. 2012). Furthermore, attending SPE may result in less contact and interactions with peers living in the neighbourhood who attend RPE. This may contribute to social barriers related to participation in inclusive outdoor play (Finnvold 2018; MacMillan et al. 2013).

To our knowledge, no study has explored perspectives from children without disabilities and children with disabilities in RPE and SPE about inclusive outdoor play (Van Engelen et al. 2021). Therefore, the research question posed in this study is as follows: ‘What facilitators and barriers are experienced by children aged 6–12 years, without disabilities and children with disabilities in RPE and SPE, during inclusive outdoor play?’

## 2 Methods

### 2.1 Research Design

This study has an exploratory qualitative design. We used an inductive thematic approach to explore facilitators and barriers perceived by Dutch children with and without disabilities related to inclusive outdoor play (Braun and Clarke 2006, 2019; Thomas et al. 2014). We applied the criteria from the Standards for Reporting Qualitative Research to improve transferability and transparency (O'Brien et al. 2014).

Three trained, female PPT master's students (NA, AC, KZ) conducted individual semi‐structured interviews between January 2019 and April 2020 under the supervision of two experienced female PPT senior researchers (EB, MB). We used an interview guide and a defined topic list (Kallio et al. 2016). To enhance the understanding of children, especially those unfamiliar with disabilities, a wheelchair and photos of assistive devices and various disabilities were shown during the interviews.

We conducted interviews both face‐to‐face and digitally via video calls (Clickdoc and Zoom) due to guidelines issued by the National Institute for Public Health and the Environment (RIVM) during the COVID‐19 pandemic (Rijksinstituut voor Volksgezondheid en Milieu Ministerie van Volksgezondheid, Welzijn en Sport 2019). The online conduct of interviews has proven to be reliable (Shapka et al. 2016; Vadi et al. 2016). The interviews were audio recorded. During interviews, children were free to choose whether or not to have their parents present.

### 2.2 Ethics

The Research Ethics Screening Committee in Health at the University of Applied Sciences Utrecht, the Netherlands, advised that the current study was exempt from the Medical Research Involving Human Subjects Act (file number: XXX). All participants and their parents received a standardized information letter, and informed consent was obtained from parents because all participants were under the age of 12.

### 2.3 Participants and Sampling

Participants (*n* = 40) were Dutch children aged 6–12 years (mean age 8.7, SD 1.9) with proficiency in the Dutch language. We defined three participant groups: children without disabilities (*n* = 22), children with disabilities attending RPE (*n* = 10) and children with disabilities attending SPE (*n* = 8). We recruited participants using flyers within the professional network of paediatric physical therapists (PPT's), researchers and through advertisements on social media (LinkedIn and Facebook).

We included children aged six and above. They needed to have sufficient development of autobiographical recall, cognitive competence and language capabilities necessary to participate in an interview, as indicated by their parents (Docherty and Sandelowski 1999). Because outdoor play generally decreases in children older than 12, we excluded children older than 12 years of age (Brockman et al. 2011).

To ensure heterogeneity, we selected participants through purposive sampling, focusing on the absence or presence of disabilities, diversity of disabilities (including visible and non‐visible disabilities), familiarity with children with disabilities, age, gender and participants' residency in either urban or rural areas (Boeije and Blijenbergh 2019; Hodkinson 2007; Kalyva and Agaliotis 2009).

### 2.4 Data Management and Analysis

The semi‐structured interviews were conducted until data saturation was achieved. Saturation was defined as the point at which no information about new themes was obtained during the last two interviews (Baarda et al. 2021). Each interview was transcribed verbatim and pseudonymized. One PPT researcher (RB) and one PPT master student (LK) analysed the data independently using ATLAS.ti 23 for Windows (Scientific Software Development GmbH, Berlin, Germany). We followed an inductive approach, guided by the six‐step process outlined in Braun and Clarke's reflexive thematic analysis (Braun and Clarke 2019).

To ensure trustworthiness, we applied several strategies in line with qualitative research standards (Korstjens and Moser 2017). These included investigator triangulation, peer debriefing sessions, purposive sampling to enhance credibility and diversity, and systematic documentation of analytic decisions throughout the process.

We thoroughly read the transcripts to gain familiarity with the dataset (step 1). Subsequently, we independently identified segments and generated initial codes (step 2). Through critical peer review, consensus was reached; if no consensus was achieved, a third PPT researcher was consulted (MB). Initially, codes and potential subthemes were developed separately for different sub‐groups (i.e., children with and without disabilities, attending RPE or SPE). However, as there was considerable overlap in the experiences described by the different groups, overarching main and sub‐themes were then constructed to reflect common barriers and facilitators, while also acknowledging subgroup‐specific nuances where relevant. This process was carried out by three PPT researchers (RB, EB, MB) to ensure investigator triangulation (step 3) (Korstjens and Moser 2017). Following this, we conducted a comprehensive review of the themes in four peer debriefing sessions, defining and naming the themes (steps 4–5). Finally, we selected quotes relevant to the identified themes and started the final analysis and write‐up of the report (step 6).

## 3 Results

The characteristics of the participants are presented in Table 1. The average duration of the interviews was 31 min (range 22–45 min).

**TABLE 1:** Characteristics of participants.

Child	Age	Gender	Diagnosis	Mode of mobility	Education
1	12	Girl	Spina bifida	Manual wheelchair	SPE
2	8	Boy	Emery–Dreyfuss	Electric wheelchair	SPE
3	9	Girl	Developmental delay E.C.I.	Walking, manual wheelchair	SPE
4	9	Girl	Cerebral palsy	Walking, manual wheelchair	SPE
5	6	Boy	Spinal muscular atrophy type 2	Electric wheelchair	SPE
6	9	Boy	SEPN1 myopathy	Walker, manual wheelchair, electric wheelchair	SPE
7	9	Girl	CTNNB1 syndrome	Manual wheelchair, walker	SPE
8	9	Girl	CHARGE syndrome	Walking, manual wheelchair	SPE
9	12	Girl	Congenital myopathy	Electric wheelchair	RPE
10	12	Boy	Artrogryposis multiplex congenita	Walking	RPE
11	6	Girl	Osteogenesis imperfecta	Walking	RPE
12	7	Boy	Cerebral palsy	Walking	RPE
13	8	Boy	Developmental coordination disorder	Walking	RPE
14	8	Girl	Erb's paly	Walking	RPE
15	7	Boy	Developmental coordination disorder	Walking	RPE
16	8	Boy	Visual impairment	Walking	RPE
17	8	Boy	Duchenne	Walking, manual wheelchair	RPE
18	6	Boy	Spinal muscular atrophy type 2	Electric wheelchair	RPE
19	10	Girl	Without disability	Walking	RPE
20	9	Boy	Without disability	Walking	RPE
21	10	Boy	Without disability	Walking	RPE
22	12	Boy	Without disability	Walking	RPE
23	6	Girl	Without disability	Walking	RPE
24	8	Girl	Without disability	Walking	RPE
25	12	Girl	Without disability	Walking	RPE
26	6	Boy	Without disability	Walking	RPE
27	8	Girl	Without disability	Walking	RPE
28	8	Boy	Without disability	Walking	RPE
29	7	Boy	Without disability	Walking	RPE
30	10	Girl	Without disability	Walking	RPE
31	10	Boy	Without disability	Walking	RPE
32	10	Boy	Without disability	Walking	RPE
33	12	Boy	Without disability	Walking	RPE
34	6	Boy	Without disability	Walking	RPE
35	7	Boy	Without disability	Walking	RPE
36	11	Girl	Without disability	Walking	RPE
37	9	Girl	Without disability	Walking	RPE
38	10	Girl	Without disability	Walking	RPE
39	7	Girl	Without disability	Walking	RPE
40	8	Girl	Without disability	Walking	RPE
	*Mean (years) 8.7* *SD (years) 1.9*				

Abbreviations: CTNNB1, CTNNB1‐gene; E.C.I., E cause ignota (unknown cause); RPE, regular primary education; SD, standard deviation; SEPN1, SEPN1‐gene; SPE, special primary education.

All children experience factors that either facilitate or act as barriers to inclusive outdoor play. While some factors are similar, notable differences are indicated by children without and with disabilities in both RPE and SPE. Children with disabilities in SPE experience the most barriers.

**FIGURE 1:**
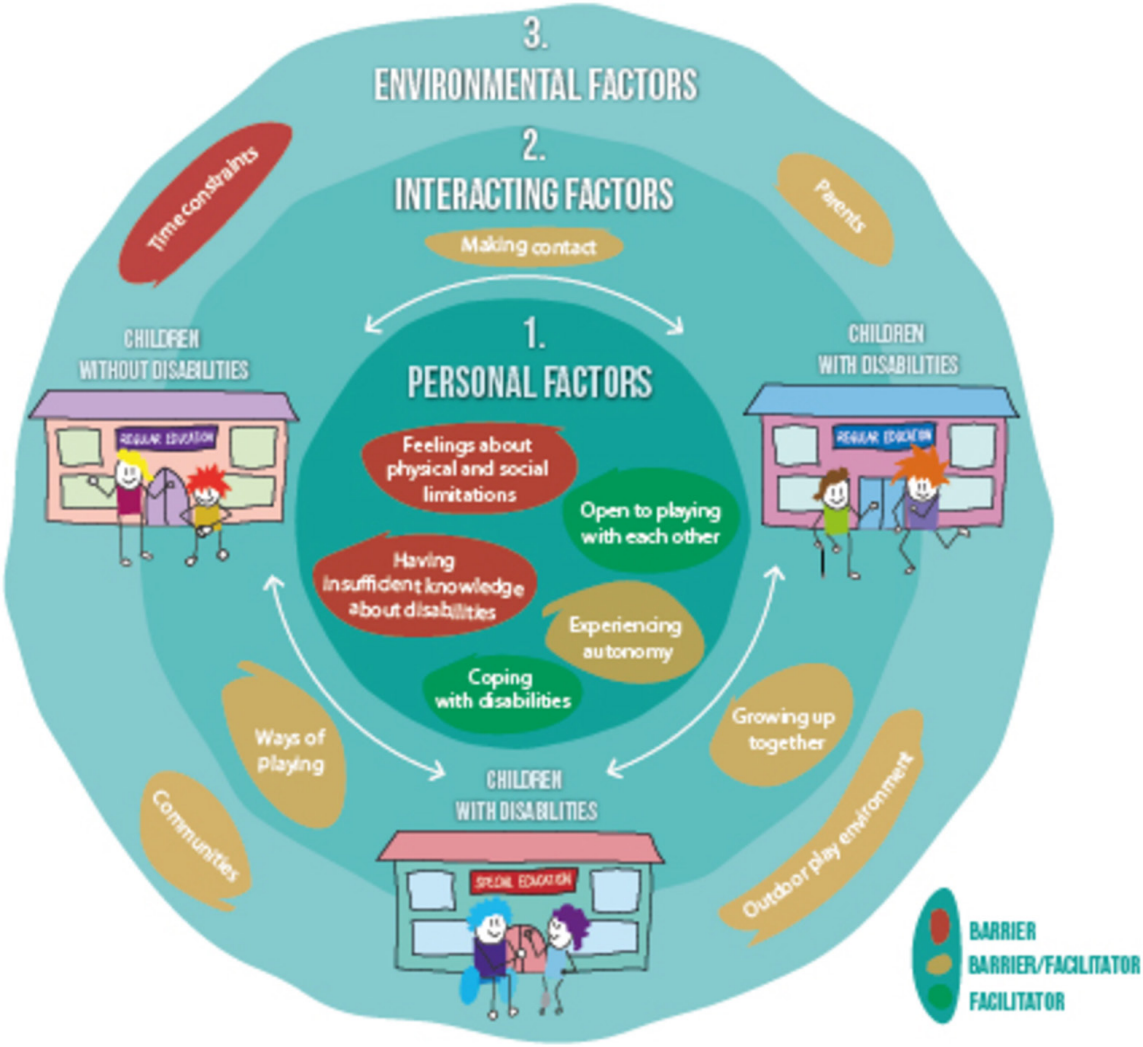
Three main themes: (1) Personal factors of children with and without disabilities, (2) Interacting factors and (3) Environmental factors. Sub‐themes are divided into facilitators and barriers. Note: In this illustration, some children are represented using assistive devices to indicate disability. We acknowledge that disabilities can be both visible and non‐visible. Our study included children with a broad range of disabilities.

We will first provide an overview of the context of outdoor play as perceived by participating children. This is essential because the quantity, location and companions with whom children engage in outdoor play shape their experiences during outdoor activities. Subsequently, we will describe facilitators and barriers for three identified main themes: (1) Personal factors of children with and without disabilities, (2) Interacting factors and (3) Environmental factors. Each main theme will be discussed based on its respective sub‐themes (Figure 1).

Through selected quotes, we aim to provide insights into the perspectives of children without disabilities (WD) and children with disabilities in RPE and SPE.

### 3.1 Context of Outdoor Play

Children with and without disabilities both indicated a preference for playing outside more often. However, other children with disabilities in both RPE and SPE expressed satisfaction with their current frequency of outdoor play. When children engage in outdoor play, choices of location vary, particularly between children with and without disabilities. Children without disabilities favour playgrounds, streets, and to a lesser extent, schoolyards. In contrast, children with disabilities, particularly those in SPE, often favour their schoolyard and prefer playing in their own garden.

Regarding play companions, children without disabilities indicate playing outside with neighbourhood children, classmates, peers and friends. Children with disabilities in RPE mention playing with classmates, friends, other children with disabilities, and to a lesser extent with neighbourhood children. Children with disabilities in SPE mainly play with classmates in SPE. Additionally, many of these children mention play with siblings and adult family members. While some also know children without disabilities in their neighbourhood and occasionally play with them, others find this more challenging.

Children with disabilities, especially those in SPE, indicate that they often play with adults or under adult supervision. While some are accustomed to it, others prefer playing without adult oversight. Children without disabilities less frequently mentioned playing outside with adult supervision.

### 3.2 Personal factors of children with and without disabilities

**FIGURE 2:**
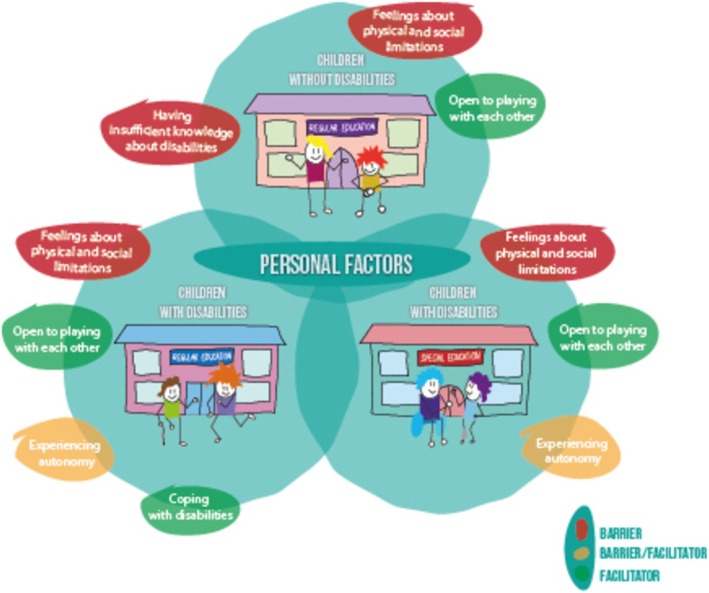
Main theme: Personal factors and its sub‐themes indicated by children with and without disabilities in regular (RPE) and special primary education (SPE). Note: In this illustration, some children are represented using assistive devices to indicate disability. We acknowledge that disabilities can be both visible and non‐visible. Our study included children with a broad range of disabilities.

Under this main theme, there are five sub‐themes that reflect the similarities and differences in the personal facilitating factors and barriers experienced by children with and without disabilities in RPE and SPE (Figure 2).

#### 3.2.1 Open to Playing With Each Other

All children express openness to playing with each other, facilitating inclusive outdoor play. Children without disabilities show interest in the nature of disabilities and are willing to assist if needed. They are open to forming friendships with children with disabilities and indicate that kindness is crucial in this regard: ‘If he's kind and all, then I could definitely be friends with him. (WD)’ Additionally, they emphasize their efforts to see the person rather than the disability. One child expressed: ‘Because those people exist too, and they can also be very nice. They just have something different, but that doesn't mean they are always annoying. They can also be very nice and fun to play with. (WD)’.

Just like children without disabilities, children with disabilities in RPE express openness to playing together outdoors. A boy responds: ‘Yes, of course! Why not! (RPE)’ to the question if he would play with other children with disabilities. Some children with disabilities in RPE mention that playing with other children with disabilities helps them develop skills in using their own assistive devices on the playground, while playing with children without disabilities encourages them to explore alternative play activities.

Children with disabilities in SPE indicate that they are open to playing outdoors with children without disabilities but mention that this occurs infrequently due to limited contact with children from their neighbourhood.

#### 3.2.2 Coping With Disabilities

Having a positive coping style is a facilitator for inclusive outdoor play. Most children with disabilities in RPE seem to approach their disabilities positively and indicate that outdoor play is possible for them by accepting their limitations, playing to the best of their abilities and utilising possibilities their disability offers. They take initiative in choosing outdoor play activities in which they can participate.

#### 3.2.3 Experiencing Autonomy

Children with disabilities are motivated to go to playgrounds independently despite facing physical limitations such as fatigue while playing. Children with disabilities in RPE feel proud when they play independently and use playground equipment without assistance. They express annoyance when praised with ‘well done’ for simple tasks. When necessary, children with disabilities in RPE and SPE are open to accept assistance from other children and parents, such as when they encounter difficulties performing transfers to use playground equipment.

#### 3.2.4 Having Insufficient Knowledge About Disabilities

A barrier to inclusive outdoor play is insufficient knowledge about disabilities and their associated capabilities and limitations. Children without disabilities indicate that they have limited interactions with children who have disabilities, leaving them unaware of why a child might use a wheelchair or the specific nature of various disabilities. Parents of children with disabilities can provide explanations, but they are not always known and present at the playground: ‘Sometimes yes and sometimes no, because sometimes I know the parents of one child, but for the other, I do not know. (WD)’ This makes it difficult for them to determine what a child can or cannot do, and they indicate that this can lead to the exclusion of children with disabilities: ‘First, they start excluding him a little because they have never encountered someone like him before. And then they think he cannot do anything at all. (WD)’.

#### 3.2.5 Feelings About Physical and Social Limitations

Having negative feelings and expectations about physical and social limitations of children with disabilities acts as a barrier to inclusive outdoor play. Children without disabilities believe that children with disabilities often struggle with outdoor play due to limited opportunities to participate, and they feel sorry for them: ‘… .. Um, yeah, I feel kind of sorry for them, you know, that they have that. If they are in a wheelchair or using a walker and then they cannot really do their thing very well anymore. (WD)’ Moreover, they think that children with disabilities are likely to face bullying during outdoor activities: ‘… .., because they are different from the others. And maybe that's why they also get bullied. (WD)’.

Children with disabilities in RPE express negative feelings about their own limitations in outdoor play activities. These limitations stem from physical disabilities and playground accessibility issues, including limited freedom to visit playgrounds at will: ‘… .., that I can just go to the playground whenever I want … and that I do not have to use a walker. (RPE)’.

Children with disabilities in SPE also have negative feelings, expressing uncertainty and anxiety about joining other children in outdoor activities. Moreover, they experience loneliness because their friends are often not present at local playgrounds, and joining other children is too much for them: ‘….. I sometimes feel upset because I am often left alone. (SPE)’.

### 3.3 Interacting factors

This main theme has three sub‐themes that indicate the facilitating factors and barriers for children with and without disabilities to interact with each other.

#### 3.3.1 Making Contact

Children decide whom to play with based on behaviour, appearance and gender of potential playmates. Children without disabilities express willingness to play with children with disabilities if they show kindness, seem lonely, or when they themselves are feeling bored: ‘It depends. I would, of course, play with a small cute girl, that's fine. But a boy acting tough… (WD)’ Children with disabilities in RPE and SPE, mention that it is also important that other children are not too hyperactive. Hyperactive behaviour results in sensory overload and fatigue and it inhibits making contact: ‘Always so busy!….. At the end of the day, I'm always very tired too. (SPE)’.

Additionally, children without disabilities and children with disabilities in RPE indicate the importance of familiarity among children during outdoor play. Some children without disabilities struggle to approach those with disabilities not because of the disability, but due to their unfamiliarity. While blending into existing groups presents another barrier for children without disabilities. They express that being approached by established groups can be intimidating, preferring to initiate contact with individuals: ‘Well, of course I don't know their reaction, so then I'm a little scared. Because if there are several, I don't like it when they approach me, I just find that scary. (WD)’.

#### 3.3.2 Growing Up Together

Growing up together, facilitates contact between children with and without disabilities and creates opportunities to play together outdoors. Some children without disabilities say they know children with disabilities, and it just takes time to get used to each other. Eventually, they better understand the consequences of the disability: ‘… .. At first, I would find that strange, but if, for example, we are in 5th grade and all the way to 8th grade, then I wouldn't find it strange anymore. (WD)’.

Children with disabilities in SPE indicate that they know children without disabilities in their neighbourhood but only occasionally play with them. Friendships with children without disabilities often stem from long‐term familiarity, with many having known each other since they were young children. A girl mentions: ‘My best friend doesn't have a disability … .. Well, I've been in class with her in preschool. (SPE)’.

#### 3.3.3 Ways of Playing

Children with and without disabilities express enjoyment in spending time together without participating in specific structured activities. For instance, they find pleasure in walking or moving around and having conversations together at playgrounds. They also acknowledge that variety in play is important to continue playing together.

Collaboration in creating and playing games fosters inclusive play experiences. It is crucial that the games played are challenging for all children. Children without disabilities mention that they can adjust their behaviour during play, such as striving for equitable play, not excluding children, and refraining from being unkind or bullying. They can also come up with creative ways to adapt the game for children with disabilities: ‘Then we could pretend she has a broken leg and that's why she's using a walker, and then we can just play with the walker. (WD)’ However, other children without disabilities indicate that they find it difficult to come up with a game or adapt one so they can play together with a child with a disability. One child says: ‘It's just a bit tricky. Yeah, I'm willing to try, but if it doesn't work, then I don't know what I can do. (WD)’.

### 3.4 Environmental factors

This main theme has four sub‐themes that indicate the environmental factors perceived as facilitating or as barriers to inclusive outdoor play.

#### 3.4.1 Parents

According to children of all groups, parents play a key role in promoting inclusive outdoor play. Particularly, children with disabilities indicate that parents are important in encouraging participation. Some children with disabilities, both in RPE and SPE, struggle with questions about their disability and prefer parental assistance in explaining their disability to other children and how to cope with it. Some of these children also express a desire for independence during outdoor play, expressing the importance of parents allowing them freedom. They prefer their parents not accompanying them to the playground, while others find it acceptable for parents to be present at a suitable distance.

Furthermore, children with disabilities in RPE and SPE emphasize the importance of being allowed by their parents to play outdoors. Bad weather conditions may prevent this permission from being granted: ‘I even prefer going outside… even if it is just a bit rainy… just a few drops, but my parents say mm (shakes head indicating no). (RPE)’.

#### 3.4.2 Outdoor Play Environment

An attractive and challenging outdoor play environment is important for all children, and elements like nature or the presence of animals motivate outdoor play. A playground should encourage cooperative play while also offering opportunities for independent or parallel play to accommodate individual preferences and needs.

Accessibility of playgrounds is of major importance. Children using assistive devices often encounter accessibility barriers at playgrounds due to narrow pathways, soft and non‐accessible surfaces such as grass, or barriers like fences impassable by wheelchairs. Additionally, children with disabilities express that numerous adapted playgrounds are situated at considerable distances from their residences, making independent access impossible.

Children without disabilities and with disabilities from RPE state that especially large playgrounds are fun to go to and that it is important that playgrounds are both physically and socially safe for children. A playground should not be too close to a busy road. Furthermore, children with disabilities from RPE have experienced that social safety at playgrounds can be disrupted by disputes between children and that social safety can be facilitated through adult supervision.

#### 3.4.3 Communities

For children with disabilities, the context in which they grow up plays an important role in inclusive outdoor play. Children with disabilities indicate that in small communities, such as in a village or school, other children and parents generally perceive their disability as normal. Children experience acceptance, encounter fewer inquiries about their disabilities and forge stronger connections with peers. According to both children without and with disabilities in RPE, their school provides a place for interaction, helping them become familiar with their schoolmates disabilities: ‘Yes, I would appreciate that because then we can learn how to interact with each other. (WD)’ However, children with disabilities in RPE often express being the only one with a disability in their class and would like to see this change.

#### 3.4.4 Time Constraints

Children with disabilities in RPE cite time constraints as a barrier for outdoor play, often associated with after‐school care. Similarly, children with disabilities in SPE note that they have limited outdoor playtime, often due to arriving home late after school because their school is not located in their residential area. They often use taxis for transportation, leading to a delayed arrival home compared to children in RPE: ‘Usually, we are in the taxi for quite a while. And of course, we cannot really ask the driver, “Hey Driver, can you drive to my friend's place”. (SPE)’.

## 4 Discussion

The purpose of this study was to explore facilitators and barriers perceived by Dutch children aged 6–12 years, without disabilities and children with disabilities in RPE and SPE for participating in inclusive outdoor play. We identified three main themes: personal factors of children with and without disabilities, interacting factors and environmental factors. Each main theme contains sub‐themes. The personal factors of children with and without disabilities are subdivided into the following: open to playing with each other, coping with disabilities, experiencing autonomy, having insufficient knowledge about disabilities and feelings about disabilities. The interacting factors include: making contact, growing up together and ways of playing. The environmental factors include parents, outdoor play environment, communities and time constraints.

We found an overlap in facilitators and barriers described by all children. Children with disabilities encounter more barriers than children without disabilities, particularly those in SPE facing most barriers.

Our results show similarities with studies focussing on adult perspectives. Children also indicate that while physical barriers like inaccessible or unplayable playgrounds hinder inclusive outdoor play, social factors such as exclusion by peers, lack of friends and the inability to adapt outdoor play behaviour have an even greater impact (Van Engelen et al. 2021; Morgenthaler et al. 2023).

### 4.1 Personal Factors of Children With and Without Disabilities

It is notable that all children are open to inclusive outdoor play and emphasise that the personality of a child is more important than their disability. However, children from all groups indicate that they have negative feelings about physical and social limitations during inclusive outdoor play.

Children with disabilities in SPE experience uncertainty and fear when participating, unlike most children with disabilities in RPE. This is linked to smaller and more segregated social networks, and fewer opportunities to interact with children without disabilities. Additionally, several children mentioned the role of parental supervision, which may unintentionally reinforce dependency and limit autonomy. Children with disabilities in RPE find it bothersome when they cannot fully participate due to their disability.

Children without disabilities often express pity toward children with disabilities, perceiving them to struggle with outdoor play, a perception that appears to stem primarily from a lack of knowledge and limited experience, rather than intentional stigma or discrimination. Although showing pity is perceived as a positive trait by children without disabilities, this perception can impact the well‐being of individuals with disabilities, disempowering them and leading to social exclusion (Armstrong et al. 2015; Babik and Gardner 2021).

Paediatric rehabilitation professionals can play a crucial role in reducing perceived barriers for children with and without disabilities. Based on our findings, their role includes providing knowledge to children and their parents about the consequences of disabilities, informing them about possible adaptations and demonstrating what children with disabilities are capable of. Moreover, they can empower children with disabilities by encouraging them to think about solutions themselves and fostering self‐efficacy (Bolster et al. 2021). Furthermore, they can facilitate positive experiences during inclusive outdoor play.

### 4.2 Interacting Factors

Children without disabilities indicate that unfamiliarity is more important than the presence of disabilities when making contact. Additionally, children with disabilities in SPE experience more and different barriers in interaction compared to children with disabilities in RPE. Children with disabilities in SPE have fewer interactions with children without disabilities. They more frequently form friendships with peers who also have disabilities, primarily classmates who do not live in the neighbourhood. As a result, children with disabilities in SPE often have fewer friendships with peers living nearby, leading to social isolation and segregation rather than fostering inclusion (Göransson et al. 2020). This finding is consistent with previous research indicating that children with disabilities in RPE have a larger social circle than those in SPE. Their social networks are more diverse and broader (Baker and Donelly 2001; Boezaard et al. 2018).

Growing up together appears to facilitate engagement in play with each other, and paediatric rehabilitation professionals can support children with disabilities to meet other children with and without disabilities at a young age. They can encourage interaction between children in the neighbourhood by providing therapy in the daily living situation of children with disabilities.

Informing and empowering parents on how to facilitate inclusive play seems important. In line with the results from Van Engelen et al. (2021), this may lead to more interaction and friendships between children with and without disabilities.

### 4.3 Environmental Factors

Some children with disabilities prefer playing independently, while others value parental presence with freedom to play and socialise and thus experience autonomy. However, it can be challenging for parents to let go of their children, making the experience of autonomy complex (Bolster et al. 2021; Van Engelen et al. 2021). Parents can support their children by sharing knowledge about the consequences of disabilities, and paediatric rehabilitation professionals can assist parents in this role by providing coaching. Furthermore, parents can empower their children to engage in outdoor play. If paediatric rehabilitation professionals shift their treatment approach to the playground context, they can identify experiences that contribute to children's autonomy. This approach can also change parents' perspectives and serve as an example.

An attractive, accessible and physically safe outdoor play environment is important for all children. However, the presence of attractive adapted play areas in the neighbourhood is often still an exception. In 2016, the UN Convention on the Rights of Persons with Disabilities came into effect in the Netherlands, stating that there should be no discrimination and that efforts should be made toward an inclusive society (Convention on the Rights of Persons with Disabilities 2016). This highlights the responsibility to provide children with disabilities equal access to participation in play and calls for action that designing and implementing inclusive play areas should become the norm. Paediatric rehabilitation professionals can collaborate with welfare professionals and policymakers to adapt playgrounds for children with disabilities. They are experts in physical activity, adapted physical activity and functioning of children and can use this expertise to help design challenging, enjoyable and inclusive play opportunities for all children.

### 4.4 Strengths and Limitations

A strength of our study is that due to the organisation of segregated education in the Netherlands, we examined perspectives of children without disabilities and children with disabilities in both RPE and SPE regarding inclusive outdoor play. To our knowledge, these different perspectives have not been collectively explored before (Van Engelen et al. 2021; Morgenthaler et al. 2023).

Interviewing young children without disabilities about inclusive outdoor play proved challenging due to their limited familiarity with the topic. To enhance their understanding, we showed photos of assistive devices and had a wheelchair present during the interviews. Furthermore, there is a possibility that children provided socially desirable answers due to the sensitivity of the topic. To address this, we opted for a large sample (*n* = 40) of participants to minimise the impact on our data.

Due to COVID‐19, we conducted both face‐to‐face interviews and online interviews, which may have resulted in shorter answers and less contextual information (Davies et al. 2020; Mirick and Wladkowski 2019). However, an online approach also may have made children feel more comfortable in their own environment. We believe that the online interviews did not influence our participants' answers, given the saturation of our data.

A limitation is that we lacked detailed clinical data on disability severity and used school placement and assistive device use as general indicators. This approach may not fully capture differences in severity that could affect children's experiences.

In this study, we explored children's perspectives regarding inclusive outdoor play. However, we did not investigate how children with and without disabilities actually engage in inclusive outdoor play. Observational research on the inclusive outdoor play behaviour of children could provide additional information about facilitating factors and barriers. Additionally, future research can focus on exploring what children need to overcome barriers and what facilitating factors for inclusive play are after experiencing playing together outdoors.

## 5 Conclusion

Children with and without disabilities in regular and special primary education indicate facilitators and barriers in personal, interacting and environmental factors for inclusive outdoor play. Personal factors include being open to playing with each other, coping with disabilities, experiencing autonomy, insufficient knowledge about disabilities and feelings about physical and social limitations. Interacting factors involve growing up together, making contact and adapting ways of playing. Parents, play environments, communities and time constraints are important environmental factors. Paediatric rehabilitation professionals, with parental support, play a crucial role in fostering inclusive outdoor play for children. This includes providing therapy in daily living situations, facilitating inclusive play experiences early on, educating about disability impacts, showcasing the capabilities of children with disabilities and collaborating to adapt playgrounds.

## Author Contributions

I have included a cover letter explaining the contribution of each author, as there are more than five authors. All authors have made an active contribution to the conception and design, and/or analysis and interpretation of the data, and/or drafting of the paper. Additionally, all authors critically reviewed its content and have approved the final version submitted for publication. Their main contributions to the manuscript are described as follows: Ryan Quint Beekhuizen MSc.: substantial contribution to conception and design of the study, data analysis, data interpretation, drafting and revising the manuscript, and final approval of the version to be published; Dr. Eline Bolster: substantial contribution to conception and design of the study, data analysis, data acquisition and interpretation, drafting and revising the manuscript, and final approval of the version to be published; Prof. dr. Jan Willem Gorter: contribution to conception and design of the study, data analysis, data interpretation, drafting and revising the manuscript, and final approval of the version to be published; Nandine Henry MSc.: contribution to data analysis, data interpretation, drafting and revising the manuscript, and final approval of the version to be published; Dr. Kirsten Visser: contribution to data analysis, data interpretation, drafting and revising the manuscript, and final approval of the version to be published; Dr. Harriet Wittink: contribution to data analysis, data interpretation, drafting and revising the manuscript, and final approval of the version to be published; Dr. Elles Kotte: contribution to data analysis, data interpretation, drafting and revising the manuscript, and final approval of the version to be published; Dr. Marleen Sol: contribution to data analysis, data interpretation, drafting and revising the manuscript, and final approval of the version to be published; Dr. Manon Bloemen: substantial contribution to conception and design of the study, data analysis, data acquisition and interpretation, drafting and revising the manuscript, and final approval of the version to be published.

## Ethics Statement

The Research Ethics Screening Committee in Health at the University of Applied Sciences Utrecht, the Netherlands, advised that the current study was exempt from the Medical Research Involving Human Subjects Act (file number: 80_000_2018).

## Conflicts of Interest

The authors declare no conflicts of interest.

## Data Availability

The data that support the findings of this study are available on request from the corresponding author. Since these are verbatim transcripts of individuals who may be identifiable, the data are not publicly available due to privacy or ethical considerations.

## References

[cch70154-bib-0001] Armstrong, M. , C. Morris , C. Abraham , O. C. Ukoumunne , and M. Tarrant . 2015. “Children's Contact With People With Disabilities and Their Attitudes Towards Disability: A Cross‐Sectional Study.” Disability and Rehabilitation 38, no. 9: 879–888. 10.3109/09638288.2015.1074727. 26289369

[cch70154-bib-0002] Baarda, B. , E. Bakker , M. Julsing , T. Fischer , V. Peters , and T. van der Velden . 2021. *Basisboek kwalitatief onderzoek: Handleiding voor het opzetten en uitvoeren van kwalitatief onderzoek* (5de dr.). Noordhoff.

[cch70154-bib-0003] Babik, I. , and E. S. Gardner . 2021. “Factors Affecting the Perception of Disability: A Developmental Perspective.” Frontiers in Psychology 12: 702166. 10.3389/fpsyg.2021.702166. 34234730 PMC8255380

[cch70154-bib-0004] Baker, K. , and M. Donelly . 2001. “The Social Experiences of Children With Disability and the Influence of Environment: A Framework for Intervention.” Disability & Society 16, no. 1: 71–85. 10.1080/713662029.

[cch70154-bib-0005] Barron, C. , A. Beckett , M. Coussens , et al. 2016. Barriers to Play and Recreation for Children and Young People with Disabilities. De Gruyter Open. 10.1515/9783110526042.

[cch70154-bib-0006] Boeije, H. , and I. Blijenbergh . 2019. *Analyseren in kwalitatief onderzoek* (3de dr.). Boom Uitgevers Amsterdam.

[cch70154-bib-0007] Boezaard, G. , M. Haitsma , and D. Nieuwenhuis . 2018. “School en vriendschappen.” https://iederin.nl/wp‐content/uploads/2019/02/1403_School__Vriendschappen_2018.pdf.

[cch70154-bib-0008] Bolster, E. A. M. , C. Van Gessel , M. Welten , et al. 2021. “Using a Co‐Design Approach to Create Tools to Facilitate Physical Activity in Children With Physical Disabilities.” Frontiers in Rehabilitation Sciences 2: 707612. 10.3389/fresc.2021.707612. 36188842 PMC9397745

[cch70154-bib-0009] Braun, V. , and V. Clarke . 2006. “Using Thematic Analysis in Psychology.” Qualitative Research in Psychology 3, no. 2: 77–101. 10.1191/1478088706qp063oa.

[cch70154-bib-0010] Braun, V. , and V. Clarke . 2019. “Reflecting on Reflexive Thematic Analysis.” Qualitative Research in Sport, Exercise and Health 11, no. 4: 589–597. 10.1080/2159676x.2019.1628806.

[cch70154-bib-0011] Brockman, R. , R. Jago , and K. R. Fox . 2011. “Children's Active Play: Self‐Reported Motivators, Barriers and Facilitators.” BMC Public Health 11, no. 1: 461. 10.1186/1471-2458-11-461. 21663605 PMC3124432

[cch70154-bib-0012] Burghard, M. , N. B. De Jong , S. Vlieger , and T. Takken . 2018. “2017 Dutch Report Card+: Results From the First Physical Activity Report Card Plus for Dutch Youth With a Chronic Disease or Disability.” Frontiers in Pediatrics 6: 122. 10.3389/fped.2018.00122. 29761094 PMC5937055

[cch70154-bib-0013] Convention on the Rights of Persons with Disabilities . 2016. https://wetten.overheid.nl/BWBV0004045/2016‐07‐14.

[cch70154-bib-0014] Davies, L. , K. L. LeClair , P. Bagley , et al. 2020. “Face‐to‐Face Compared With Online Collected Accounts of Health and Illness Experiences: A Scoping Review.” Qualitative Health Research 30, no. 13: 2092–2102. 10.1177/1049732320935835. 32667257

[cch70154-bib-0015] de Vries, S. 2012. “Belang van Buitenspelen.” Literatuurstudie naar de gezondheidswaarde, de sociale en de economische waarde. https://www.documentatiecentrum.nl.

[cch70154-bib-0016] Docherty, S. , and M. Sandelowski . 1999. “Focus on Qualitative Methods: Interviewing Children.” Research in Nursing & Health 22, no. 2: 177–185. 10.1002/(SICI)1098-240X(199904)22:2<177::AID-NUR9>3.0.CO;2-H. 10094302

[cch70154-bib-0017] Donnelly, J. E. , C. H. Hillman , D. Castelli , et al. 2016. “Physical Activity, Fitness, Cognitive Function, and Academic Achievement in Children.” Medicine & Science in Sports & Exercise 48, no. 6: 1197–1222. 10.1249/mss.0000000000000901. 27182986 PMC4874515

[cch70154-bib-0018] Eime, R. M. , J. A. Young , J. T. Harvey , M. J. Charity , and W. R. Payne . 2013. “A Systematic Review of the Psychological and Social Benefits of Participation in Sport for Adults: Informing Development of a Conceptual Model of Health Through Sport.” International Journal of Behavioral Nutrition and Physical Activity 10, no. 1: 135. 10.1186/1479-5868-10-135. 24313992 PMC4028858

[cch70154-bib-0019] Finnvold, J. E. 2018. “School Segregation and Social Participation: The Case of Norwegian Children With Physical Disabilities.” European Journal of Special Needs Education 33, no. 2: 187–204. 10.1080/08856257.2018.1424781.

[cch70154-bib-0020] Fjørtoft, I. 2001. “The Natural Environment as a Playground for Children: The Impact of Outdoor Play Activities in Pre‐Primary School Children.” Early Childhood Education Journal 29, no. 2: 111–117. 10.1023/A:1012576913074.

[cch70154-bib-0021] Gill, T . 2014. “The Play Return: A Review of the Wider Impact of Play Initiatives.” https://hub.careinspectorate.com/media/1388/the‐play‐return‐a‐review‐of‐the‐wider‐impact‐of‐play‐initiatives.pdf.

[cch70154-bib-0022] Göransson, K. , K. Bengtsson , S. Hansson , N. Klang , G. Lindqvist , and C. Nilholm . 2020. “Segregated Education as a Challenge to Inclusive Processes: A Total Population Study of Swedish Teachers' Views on Education for Pupils With Intellectual Disability.” International Journal of Inclusive Education 26, no. 14: 1367–1382. 10.1080/13603116.2020.1810789.

[cch70154-bib-0023] Hodkinson, A. 2007. “Inclusive Education and the Cultural Representation of Disability and Disabled People: Recipe for Disaster or Catalyst of Change?” Research in Education 77, no. 1: 56–76. 10.7227/rie.77.5.

[cch70154-bib-0046] International Play Association . 2016. “Children’s Right to Play and the Environment.” A Discussion Paper Prepared by the International Play Association: Promoting the Child’s Right to Play for the UN Committee on the Rights of the Child Day of General Discussion. Retrieved from https://ipaworld.org/wp‐content/uploads/2016/05/IPA‐Play‐Environment‐Discussion‐Paper.pdf.

[cch70154-bib-0024] Janssen, I. , and A. G. Leblanc . 2010. “Systematic Review of the Health Benefits of Physical Activity and Fitness in School‐Aged Children and Youth.” International Journal of Behavioral Nutrition and Physical Activity 7, no. 1: 40. 10.1186/1479-5868-7-40. 20459784 PMC2885312

[cch70154-bib-0025] Kaal, M . 2022. “Onderzoek buitenspelen 2022.” https://jantjebeton.nl/uploads/downloads/onderzoek‐buitenspelen‐2022‐62a06183eb380.pdf.

[cch70154-bib-0026] Kallio, H. , A. Pietilä , M. Johnson , and M. Kangasniemi . 2016. “Systematic Methodological Review: Developing a Framework for a Qualitative Semi‐Structured Interview Guide.” Journal of Advanced Nursing 72, no. 12: 2954–2965. 10.1111/jan.13031. 27221824

[cch70154-bib-0027] Kalyva, E. , and I. Agaliotis . 2009. “Can Contact Affect Greek Children's Understanding of and Attitudes Towards Peers With Physical Disabilities?” European Journal of Special Needs Education 24, no. 2: 213–220. 10.1080/08856250902793701.

[cch70154-bib-0028] Keawutan, P. , K. Bell , P. S. Davies , and R. N. Boyd . 2014. “Systematic Review of the Relationship Between Habitual Physical Activity and Motor Capacity in Children With Cerebral Palsy.” Research in Developmental Disabilities 35, no. 6: 1301–1309. 10.1016/j.ridd.2014.03.028. 24694659

[cch70154-bib-0029] Korstjens, I. , and A. Moser . 2017. “Series: Practical Guidance to Qualitative Research. Part 4: Trustworthiness and Publishing.” European Journal of General Practice 24, no. 1: 120–124. 10.1080/13814788.2017.1375092. 29202616 PMC8816392

[cch70154-bib-0030] Lankhorst, K. , T. Takken , M. Zwinkels , et al. 2021. “Sports Participation, Physical Activity, and Health‐Related Fitness in Youth with Chronic Diseases or Physical Disabilities: The Health in Adapted Youth Sports Study.” Journal of Strength and Conditioning Research 35, no. 8: 2327–2337. 10.1519/jsc.0000000000003098. 31210643

[cch70154-bib-0031] MacMillan, M. , M. Tarrant , C. Abraham , and C. Morris . 2013. “The Association Between Children's Contact With People With Disabilities and Their Attitudes Towards Disability: A Systematic Review.” Developmental Medicine and Child Neurology 56, no. 6: 529–546. 10.1111/dmcn.12326. 24219501

[cch70154-bib-0032] Maher, C. A. , M. Toohey , and M. Ferguson . 2015. “Physical Activity Predicts Quality of Life and Happiness in Children and Adolescents With Cerebral Palsy.” Disability and Rehabilitation 38, no. 9: 865–869. 10.3109/09638288.2015.1066450. 26218617

[cch70154-bib-0033] Mirick, R. , and S. Wladkowski . 2019. “Skype in Qualitative Interviews: Participant and Researcher Perspectives.” *The Qualitative Report* 10.46743/2160-3715/2019.3632.

[cch70154-bib-0034] Morgenthaler, T. , C. Schulze , D. Pentland , and H. Lynch . 2023. “Environmental Qualities That Enhance Outdoor Play in Community Playgrounds from the Perspective of Children With and Without Disabilities: A Scoping Review.” International Journal of Environmental Research and Public Health 20, no. 3: 1763. 10.3390/ijerph20031763. 36767130 PMC9913926

[cch70154-bib-0035] Nijhof, S. L. , C. H. Vinkers , S. M. Van Geelen , et al. 2018. “Healthy Play, Better Coping: The Importance of Play for the Development of Children in Health and Disease.” Neuroscience & Biobehavioral Reviews 95: 421–429. 10.1016/j.neubiorev.2018.09.024. 30273634

[cch70154-bib-0036] O'Brien, B. C. , I. B. Harris , T. J. Beckman , D. A. Reed , and D. A. Cook . 2014. “Standards for Reporting Qualitative Research.” Academic Medicine 89, no. 9: 1245–1251. 10.1097/acm.0000000000000388. 24979285

[cch70154-bib-0037] Rijksinstituut voor Volksgezondheid en Milieu Ministerie van Volksgezondheid, Welzijn en Sport . 2019. “COVID‐19 Richtlijn.” https://www.rivm.nl.

[cch70154-bib-0038] SamenSpeelAkkoord . 2019. https://www.samenspeelnetwerk.nl/media/pages/over‐samenspeelnetwerk/7181b5c27f‐1664476849/samenspeelakkoord_toeg.pdf.

[cch70154-bib-0045] Shapka, J. D. , J. F. Domene , S. Khan , and L. M. Yang . 2016. “Online Versus In‐Person Interviews With Adolescents: An Exploration of Data Equivalence.” Computers in Human Behavior 58: 361–367. 10.1016/j.chb.2016.01.016.

[cch70154-bib-0039] Thomas, A. , A. Menon , J. Boruff , A. M. Rodriguez , and S. Ahmed . 2014. “Applications of Social Constructivist Learning Theories in Knowledge Translation for Healthcare Professionals: A Scoping Review.” Implementation Science 9, no. 1: 54. 10.1186/1748-5908-9-54. 24885925 PMC4040365

[cch70154-bib-0040] United Nations General Assembly . 1989. “Convention on the Rights of the Child.” https://www.ohchr.org/sites/default/files/crc.pdf.

[cch70154-bib-0041] Vadi, M. G. , M. R. Malkin , J. Lenart , G. R. Stier , J. W. Gatling , and R. L. Applegate II. 2016. “Comparison of Web‐Based and Face‐to‐Face Interviews for Application to an Anesthesiology Training Program: A Pilot Study.” International Journal of Medical Education 7: 102–108. 10.5116/ijme.56e5.491a. 27039029 PMC4820321

[cch70154-bib-0042] Van Engelen, L. , M. Ebbers , M. Boonzaaijer , E. A. M. Bolster , E. A. H. Van Der Put , and M. A. T. Bloemen . 2021. “Barriers, Facilitators and Solutions for Active Inclusive Play for Children With a Physical Disability in the Netherlands: A Qualitative Study.” BMC Pediatrics 21, no. 1: 369. 10.1186/s12887-021-02827-5. 34454470 PMC8401178

[cch70154-bib-0043] Vanderschuren, L. , and V. Trezza . 2014. “What the Laboratory Rat Has Taught Us About Social Play Behaviour: Role in Behavioural Development and Neural Mechanisms.” Current Topics in Behavioral Neurosciences 16: 189–212. 10.1007/7854_2013_268. 24338663

[cch70154-bib-0044] Verschuren, O. , L. Wiart , D. Hermans , and M. Ketelaar . 2012. “Identification of Facilitators and Barriers to Physical Activity in Children and Adolescents With Cerebral Palsy.” Journal of Pediatrics 161, no. 3: 488–494. 10.1016/j.jpeds.2012.02.042. 22494875

